# New insights into mechanisms behind miscarriage

**DOI:** 10.1186/1741-7015-11-154

**Published:** 2013-06-26

**Authors:** Elisabeth Clare Larsen, Ole Bjarne Christiansen, Astrid Marie Kolte, Nick Macklon

**Affiliations:** 1 The Fertility Clinic, Juliane Marie Centre, Rigshospitalet, University of Copenhagen, Copenhagen, Denmark; 2 Department of Obstetrics and Gynaecology, Aalborg Hospital, Aalborg, Denmark; 3 Division of Human Development and Health, University of Southampton, Southampton, UK; 4 Obstetrics and Gynaecology, Academic Unit of Human Development and Health, University of Southampton, Southampton, UK; 5 Complete Fertility Centre Southampton, Princess Anne Hospital, Coxford Road, Southampton, SO16 5YA, UK

**Keywords:** Embryo Selection, Epidemiology, Genetics, Immunology, Miscarriage, Recurrent Miscarriage, Sperm DNA Integrity

## Abstract

Sporadic miscarriage is the most common complication of early pregnancy. Two or three consecutive pregnancy losses is a less common phenomenon, and this is considered a distinct disease entity. Sporadic miscarriages are considered to primarily represent failure of abnormal embryos to progress to viability. Recurrent miscarriage is thought to have multiple etiologies, including parental chromosomal anomalies, maternal thrombophilic disorders, immune dysfunction and various endocrine disturbances. However, none of these conditions is specific to recurrent miscarriage or always associated with repeated early pregnancy loss. In recent years, new theories about the mechanisms behind sporadic and recurrent miscarriage have emerged. Epidemiological and genetic studies suggest a multifactorial background where immunological dysregulation in pregnancy may play a role, as well as lifestyle factors and changes in sperm DNA integrity. Recent experimental evidence has led to the concept that the decidualized endometrium acts as biosensor of embryo quality, which if disrupted, may lead to implantation of embryos destined to miscarry. These new insights into the mechanisms behind miscarriage offer the prospect of novel effective interventions that may prevent this distressing condition.

## Introduction

The term ‘miscarriage’ is applied to many complications of early pregnancy, and it is important to be clear on terminology. In 2005, the European Society of Human Reproduction and Embryology (ESHRE) introduced a revised terminology regarding early pregnancy events [1]. A pregnancy loss that occurs after a positive urinary human chorionic gonadotropin (hCG) or a raised serum β-hCG but before ultrasound or histological verification is defined as a ‘biochemical loss’. In general, these occur before 6 weeks of gestation. The term clinical miscarriage is used when ultrasound examination or histological evidence has confirmed that an intrauterine pregnancy has existed. Clinical miscarriages may be subdivided into early clinical pregnancy losses (before gestational week 12) and late clinical pregnancy losses (gestational weeks 12 to 21). There is no consensus on the number of pregnancy losses needed to fulfill the criteria for recurrent miscarriage (RM), but ESHRE guidelines define RM as three or more consecutive pregnancy losses before 22 weeks of gestation [2]. Although the above-mentioned terminology is widely used, it is also acknowledged that it is not always clinically useful. Indeed, a recent paper has proposed classification according to developmental periods in gestation [3].

Clinical miscarriage is both a common and distressing complication of early pregnancy. In recent years, progress in the fields of cytogenetics and immunogenetics and a greater understanding of implantation and maternal-embryo interactions has offered new insights into the possible causes of this condition, and opened up new avenues for research into its prevention and treatment. In this article we review the key mechanisms thought to underlie miscarriage, and discuss emerging concepts in this field (Table 1).

**Table 1:** Overview of miscarriage-associated factors and their possible causal role for miscarriage/recurrent miscarriage, possible treatments and proposals for future research

**Biomarker/lifestyle factor in patients with miscarriage/recurrent miscarriage**	**Documentation for causality**	**Possible treatment and its documented effect**	**Future research**
Parental chromosome abnormalities [4,5]	Strong	PGD: weak	Identification of high risk carriers through clinical history; RCT of PGD/no PGD
Autoantibodies [6,7]	Moderate	Prednisone, IvIg: weak	RCTs of prednisone and/or IvIg
NK cell dysfunction [8-10]	Weak to moderate	Prednisone, IvIg: weak	Develop standardized methods of measuring NK cells in the endometrium; establish normal values of NK cells in the blood and endometrium during pregnancy
Abnormal HLA-G expression [11]	Weak to moderate	Prednisone, IvIg: weak	Develop standardized methods for measuring soluble and membrane-bound HLA-G
Hereditary thrombophilia [12,13]	Moderate	Heparin, LDA: weak	RCTs of heparin and LDA
Acquired thrombophilia [12,14]	Strong	Heparin, LDA: moderate	Larger RCTs of heparin and LDA
Thyroid autoimmunity [15-17]	Strong	Levothyroxine: weak	RCTs of levothyroxin
PCOS [18]	Weak	Weight loss	Cohort studies of miscarriage rates subsequent to weight loss vs no weight loss
Sperm DNA fragmentation [19,20]	Moderate	Sperm separation: no	Identify the most specific assays; establish methods for efficient sperm selection.
Disrupted endometrial selection [21-26]	Recently proposed mechanism	Correction of decidual selective phenotype by hormonal modulators, including progesterone.	Intervention studies using hormonal treatments in the early luteal phase are being carried out
Uterine malformations [27,28]	Weak to moderate	Septal resection	RCTs of septal resection/no resection
hCG gene polymorphisms [29,30]	Weak to moderate	hCG supplementation: weak	RCTs of hCG supplementation
Alcohol consumption [31]	Moderate	Alcohol cessation	NA
Obesity [32,33]	Weak to moderate	Weight loss: weak	Cohort studies of miscarriage rates subsequent to weight loss vs no weight loss

*hCG* human chorionic gonadotropin, *HLA* human leukocyte antigen, *IvIg* intravenous immunoglobulin, *LDA* low-dose aspirin, *NK* natural killer, *PCOS* polycystic ovary syndrome, *PGD* preimplantation genetic diagnosis, *RCT* randomized controlled trial.

### Epidemiology of sporadic and recurrent miscarriage

**Figure 1:**
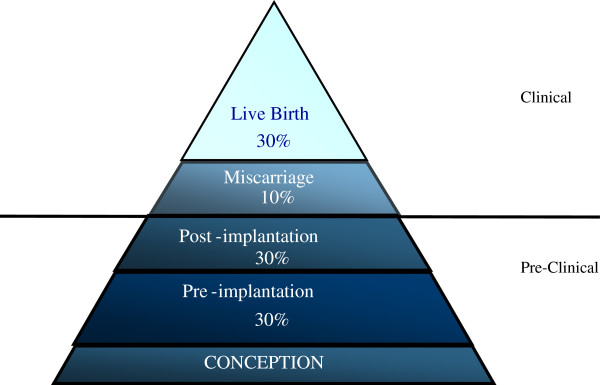
**The pregnancy loss iceberg: an overview of the outcome of spontaneous human conceptions.** It is estimated that 70% of conceptions are lost prior to live birth. The majority of these losses occur prior to implantation or before the missed menstrual period, and since they are not revealed to the woman they are termed preclinical. In the pregnancy loss ‘iceberg’, they are therefore below the ‘waterline’. Figure reproduced with permission from Oxford University Press [37].

Human reproduction is characterized by its inefficiency. Prospective cohort studies using sensitive and specific daily urinary hCG assays in women trying to conceive have demonstrated that only around one-third of conceptions progress to a live birth [34-36]. An estimated 30% of human conceptions are lost prior to implantation and a further 30% following implantation but before the missed menstrual period, that is in the third or fourth week of gestation. These are often termed preclinical losses [37] (Figure 1). Finally, the incidence of early clinical pregnancy loss is estimated to be 15% of conceptions with a significant variation according to age. Thus, the incidence ranges from 10% in women aged 20 to 24 years to 51% in women aged 40 to 44 years [38]. Late losses between 12 and 22 weeks occur less frequently and constitute around 4% of pregnancy outcomes [39].

Compared to sporadic miscarriage the prevalence of RM is considerably lower irrespective of whether biochemical losses are included or not. If only clinical miscarriages are included the prevalence is 0.8% to 1.4% [40]. If, however, biochemical losses are included the prevalence is estimated to be as high as 2% to 3%. Since the incidence of RM is greater than would be predicted by chance, it is considered to represent a disease entity defined by a series of events, with a number of possible etiologies [41].

### Mechanisms and reasons for ‘physiological’ early pregnancy loss

It is a generally accepted assumption that sporadic pregnancy losses occurring before an embryo has developed represent a ‘physiological’ phenomenon, which prevents conceptions affected by serious structural malformations or chromosomal aberrations incompatible with life from progressing to viability. This concept is supported by clinical studies in which embryoscopy was used to assess fetal morphology prior to removal by uterine evacuation. Fetal malformations were observed in 85% of cases presenting with early clinical miscarriage [42]. The same study also demonstrated that 75% of the fetuses had an abnormal karyotype. Fetal chromosomal aneuploidies arising from non-inherited and non-disjunctional events are common. Indeed, in a recent study using comparative genomic hybridization to study the chromosomal complement of all blastomeres in preimplantation human embryos, more than 90% were found to have at least one chromosomal abnormality in one or more cells [43]. The clinical implications of minor, mosaic and possibly ‘transient’ aneuploidies remain unclear. However, while most fetuses with severe developmental defects will die *in utero*[44] some aneuploidies can be compatible with survival to term. The most commonly encountered is trisomy 21, although 80% of affected embryos perish *in utero* or in the neonatal period [45]. In most cases, the extra chromosome is of maternal origin and caused by a malsegregation event in the first meiotic division. The risk of this increases with maternal age and may be considered to be a biological rather than pathological phenomenon.

Although fetal chromosomal aberrations may be identified in 29% to 60% of cases in women with RM, the incidence decreases as the number of miscarriages increases suggesting other mechanisms as a cause of the miscarriage in RM couples with multiple losses [46].

In the near future diagnostic tests on fetal genetic material isolated from maternal plasma will be a routine procedure and probably substitute chorion villus sampling and amniocentesis for prenatal diagnosis of fetal genetic diseases [47]. Today, cell-free fetal DNA can be isolated from the maternal circulation from 7 weeks of gestation, and numerous studies have already been published where next generation sequencing techniques have been applied to detect fetal aneuploidies in cell-free fetal DNA [48-50]. Since it will soon be possible to sequence the entire fetal genome from free fetal DNA in the maternal circulation, new insights will be achieved in relation to both chromosomal abnormalities and single gene disorders as a cause of sporadic and recurrent miscarriage.

### Karyotypic disorders

A chromosomal abnormality in one partner is found in 3% to 6% of RM couples, which is ten times higher than the background population [51]. The most commonly encountered abnormalities include balanced translocations and inversions that do not have any consequences for the phenotype of the carrier, but in pregnancy there is a 50% risk of a fetus with an unbalanced chromosomal abnormality that can result in a miscarriage. This risk is influenced by the size and the genetic content of the rearranged chromosomal segments. Whether or not to screen couples with RM for chromosomal abnormalities remains a topic of debate. The argument for performing this costly analysis is to optimize the counseling of RM couples with respect to any subsequent pregnancy and to avoid the birth of a child with congenital defects and mental handicaps due to an unbalanced karyotype by offering appropriate prenatal diagnostic screening.

The case against offering routine karyotyping for couples with RM rests primarily on the findings of a large index-control study with a mean follow-up period of 5.8 years. This study showed that carrier couples with at least two previous miscarriages had the same chance of having a healthy child as non-carrier couples with at least two miscarriages (83% and 84%, respectively), and more importantly a low risk (0.8%) of pregnancies with an unbalanced karyotype surviving into the second trimester [52]. Current clinical guidelines do recommend parental karyotyping as part of the evaluation in RM couples with a high risk of carrier status [4,5] but only if maternal age is low at the second miscarriage, or if there is a history of two or more miscarriages in first degree relatives [53].

Some clinicians recommend *in vitro* fertilization with preimplantation genetic diagnosis (PGD) as a treatment option in RM couples with carrier status in order to replace euploid embryos only. This may be beneficial in couples with coexisting infertility, but in couples with proven fertility the live birth rate seems to be comparable or maybe even higher after spontaneous conception including PGD [54,55].

### Immunological and immunogenetic causes

It has long been an enigma how the implanting embryo and trophoblast escape maternal immunological rejection in the uterus in spite of carrying allogeneic proteins encoded by paternal genes. A series of mechanisms regulating maternal immune recognition and fetal antigen expression has been suggested to prevent the rejection of the majority of pregnancies, but these may cause RM when they fail.

Since reproductive success is of utmost importance for the survival of a species, it is likely that redundant mechanisms have developed to prevent immune rejection of the embryo, and only when several mechanisms fail in a woman will RM will occur. This complexity continues to feed the ongoing controversy regarding which immunological factors play a role in the pathogenesis of RM.

There is general agreement that a series of autoantibodies such as anti-phospholipid, anti-nuclear and anti-thyroid antibodies can be found with increased prevalence in RM patients and may display a negative prognostic impact. However, in humans there is no proof that the antibodies *per se* harm the pregnancy; they may simply be markers of a predisposition to disruption of immunological self-tolerance and proinflammatory responses in these women. In contrast, a study found that pregnant mice injected with human IgG from a patient with anti-phospholipid antibodies significantly increased fetal resorption rate and reduced fetal weight while simultaneous treatment with antibodies blocking activation of the complement cascade completely prevented fetal resorptions and growth retardation [6]. In this and similar studies it was also found that mice deficient in various complement factors were resistant to fetal injury induced by injection of the anti-phospholipid antibodies. This indicates that at least in mice, anti-phospholipid antibodies may exercise their harmful effect on pregnancies through immunological mechanisms (complement activation) rather than through a direct procoagulant effect. There is some, however, weaker evidence that anti-phospholipid antibodies also induce complement activation in humans with antiphospholipid syndrome [7].

A series of studies have reported that increased concentrations of proinflammatory or T helper cell type I cytokines [56] or increased frequencies of subsets of natural killer (NK) cells in the blood [8] can be found during euploid sporadic miscarriage and in women with RM but it is debated whether measurements of these biomarkers in peripheral blood reflect conditions at the fetomaternal interface. There is some evidence that uterine NK cells regulate angiogenesis in the non-pregnant endometrium and therefore may also play a role for implantation and early pregnancy [9] but a systematic review of relevant studies did not find peripheral blood or uterine NK cell density or activity to be predictive for pregnancy outcome in patients with RM [10].

The most convincing evidence for the importance of the immune system in miscarriage and RM comes from genetic/epidemiologic studies showing that genetic biomarkers of possible importance for immunologic dysregulation in pregnancy are found with increased frequency in women with RM and display a negative impact on the prognosis. Examples of such genetic biomarkers are maternal homozygocity for a 14 base-pair insertion in the human leukocyte antigen (HLA)-G gene [11], maternal carriage of HLA class II alleles predisposing to immunity against male-specific minor histocompatibility antigens found on male embryos [57], specific maternal NK cell receptor genotypes in combination with fetal HLA-C genotypes that may be associated with aberrant maternal NK cell recognition of the trophoblast [58] and maternal mannose-binding lectin binding genotypes predisposing to low plasma levels of mannose-binding lectin, which may be of importance for release of cytokines and clearance of apoptopic trophoblast cells [59].

Proposed treatment options for RM where immunologic dysregulation is suggested to play a role include prednisone, allogeneic lymphocyte immunization, intravenous immunoglobulin infusion and injection of tumor necrosis factor α (TNFα) antagonists or granulocyte colony-stimulating factor (G-CSF). Much controversy exists about the efficacy of these treatments since the majority have not been subject to rigorous clinical study or have only been tested in few and small randomized controlled trials [60].

The best documented immunological treatment is intravenous immunoglobulin (IvIg), which in a recent meta-analyses in women with secondary RM was shown to improve the chance of live birth compared with placebo (OR = 1.89, 95% CI 0.93 to 3.85) [61]. However, this effect did not reach statistical significance and appropriately powered randomized controlled trials focusing on this patient subset are required to elucidate the clinical value of this therapeutic approach. In mice models there is good evidence that both unfractionated and low-molecular-weight heparin prevented complement activation and protected against pregnancy complications induced by injection of IgG from patients with antiphospholipid syndrome emphasizing the immunological effects of heparin [62].

### Thrombophilias

Thrombophilic factors predisposing to thromboembolic events are associated with both sporadic miscarriages and RM and can be hereditary or acquired [12]. It is suggested that the association is caused by an increased risk of thrombus formation in the nascent placental vessels resulting in placenta infarctions. Hereditary factors include deficiency of antithrombin, protein C and protein S or carriage of the factor V Leiden or factor II (G20210A) gene mutations. Acquired factors include the presence of anti-phospholipid antibodies, lupus anticoagulant or anti-cardiolipin antibodies, which are deemed to be present when identified in repeated samples taken 3 months apart and outwith pregnancy. Hyperhomocysteinemia can be both hereditary and acquired. There is some evidence from two non-blinded randomized controlled trials that treatment with low-dose heparin and aspirin during pregnancy increases the chance of live birth in RM patients with anti-phospholipid antibodies [14]. There is no evidence that anticoagulation therapy will improve the prognosis for RM patients with hereditary thrombophilias or no thrombophilia factors at all [13], and results from relevant ongoing randomized controlled trials are awaited (for example, the ALIFE2 study). Therapy with high-dose folate will lower plasma homocysteine levels but there is no evidence from clinical trials whether this decreases the risk of a new miscarriage.

### Endocrinological causes

The prevalence of hypothyroidism with or without underlying thyroid autoimmunity is significant among fertile women in fertile age. There is evidence that thyroid dysfunction and thyroid autoimmunity is associated with infertility and pregnancy loss both in the situation where the woman is euthyroid with thyroid antibodies and in a thyroid antibody negative woman with an elevated level of thyroid stimulating hormone (TSH) [15]. According to a recent meta-analysis of 38 studies, the presence of antibodies against thyroperoxidase (TPO-Ab) increased the risk of sporadic miscarriage with an odds ratio of 3.73 (95% CI 1.8 to 7.6) as well as RM (OR 2.3, 95% CI 1.5 to 3.5) [16]. In a large prospective study including pregnant thyroid antibody negative women, a TSH level within the normal range but higher than 2.5 mIU/L in the first trimester, nearly doubled the risk of a miscarriage [17]. However, the true significance of thyroid dysfunction and the value of its correction in improving outcomes in RM remains unclear.

Polycystic ovarian syndrome (PCOS) is a common endocrine disorder of reproductive-age women. PCOS may be associated with ovulatory disorder and miscarriage when fertility is desired. Using strict criteria the prevalence of PCOS among women with RM is estimated to be 8.3% to 10% [18]. The mechanisms behind an increased miscarriage risk in women with PCOS remains partly unclear. The current view is that the main cause may be the associated obesity, which is dealt with in the section describing lifestyle factors.

### Sperm DNA fragmentation

Sperm DNA integrity is essential to reproduction, and measurement of sperm DNA fragmentation (SDF) was therefore first introduced as an additional tool in predicting male infertility. Indeed there is a correlation between low semen quality and high SDF levels, but at present much controversy exists with regard to cut-off levels, which assay to use, and the clinical relevance of the tests in assisted reproductive technologies [63].

In contrast, there is a documented link between DNA damage in sperm and miscarriage. A recent meta-analysis including 16 studies found a highly significant increase in miscarriage rate in couples where the male partner had elevated levels of sperm DNA damage compared to those where the male partner had low levels of sperm DNA damage (risk ratio = 2.16 (1.54, 3.03, *P* <0.00001) [19]. Due to variation in study characteristics, the authors have subgrouped the included studies according to whether raw or prepared semen was analyzed, and according to which type of assay was used to determine sperm DNA damage. A consistent and significant association with miscarriage was found regardless of which semen preparation was used while the strongest association as regards assays was found in a test quantifying sperm DNA damage directly by incorporating a labeled enzyme into single and double-stranded DNA breaks.

In a study comparing fertile sperm donors with couples who have unexplained RM, an assay was used that both measured DNA damage directly and also distinguished between single-stranded and double-stranded DNA damage. The study showed that 85% of the RM couples had a profile with high values of double-stranded DNA damage compared to only 33% among fertile sperm donors, suggesting a specific paternal explanation in these otherwise unexplained cases [20].

In the future, assays detecting sperm DNA damage may be introduced into the evaluation of couples who experience RM, and in an infertility setting the development of methods that select sperm without DNA damage may be helpful in reducing the risk of miscarriage.

### Failure of embryo selection

Recent *in vitro* studies of embryo-decidual interactions have demonstrated that decidualized stromal cells act as a biosensor for embryonic derived signals and appear capable of ‘selecting’ embryos for implantation on the basis of their quality. The first study to demonstrate the biosensor function of decidualized endometrial stromal cells (ESC) showed that coculture with an arresting human embryo elicited a reduction in the production of key cytokine regulators of implantation including interleukin (IL)-1β, heparin-binding epidermal growth factor-like growth factor (HB-EGF), IL-6, and IL-10 [21]. These findings provided the first experimental evidence to support the hypothesis initially put forward by Quenby *et al*. that some women with RM may be allowing embryos of poor viability to implant inappropriately [22]. In other words, women who experience RM may not be rejecting healthy embryos, but rather permitting embryos of low viability to implant long enough to present as a clinical pregnancy before rather than being lost as a preclinical biochemical pregnancy.

The hypothesis that endometrial selectivity to embryo quality may be disrupted in women with RM has found support from a number of other studies. Women with RM have been shown to express lower levels of endometrial mucin 1, an antiadhesion molecule that contributes to the barrier function of the epithelium [23]. Moreover, ESCs of women with RM show an abnormal response to decidualization *in vitro*, manifest by attenuated prolactin (PRL) production and prolonged and enhanced prokineticin 1 expression [24]. It has been proposed that this may result in a prolonged ‘window’ of receptivity to implantation, but a reduction in the selective functions of the decidua [25].

If women with RM are less selective to embryos implanting, then it would be expected that they would report shorter intervals between pregnancies. This has indeed been demonstrated in a retrospective cohort study of 560 RM women, which showed a significantly greater proportion to have time-to-pregnancy intervals of 3 months or less compared with fertile control subjects [24].

Further experimental evidence supporting low endometrial selectivity or ‘super receptivity’ in women with RM has come from studies of stromal cell migration *in vitro*. Recently it has been shown that ESC migration occurs around the time of embryo implantation and may promote implantation by encapsulation of the conceptus [64,65]. In timelapse imaging studies covering a period of 48 h, ESC migration was clearly depicted at the site of embryo implantation. Moreover, the ESCs showed migration around the embryo suggesting an active role for ESCs in the implantation process [64].

**Figure 2:**
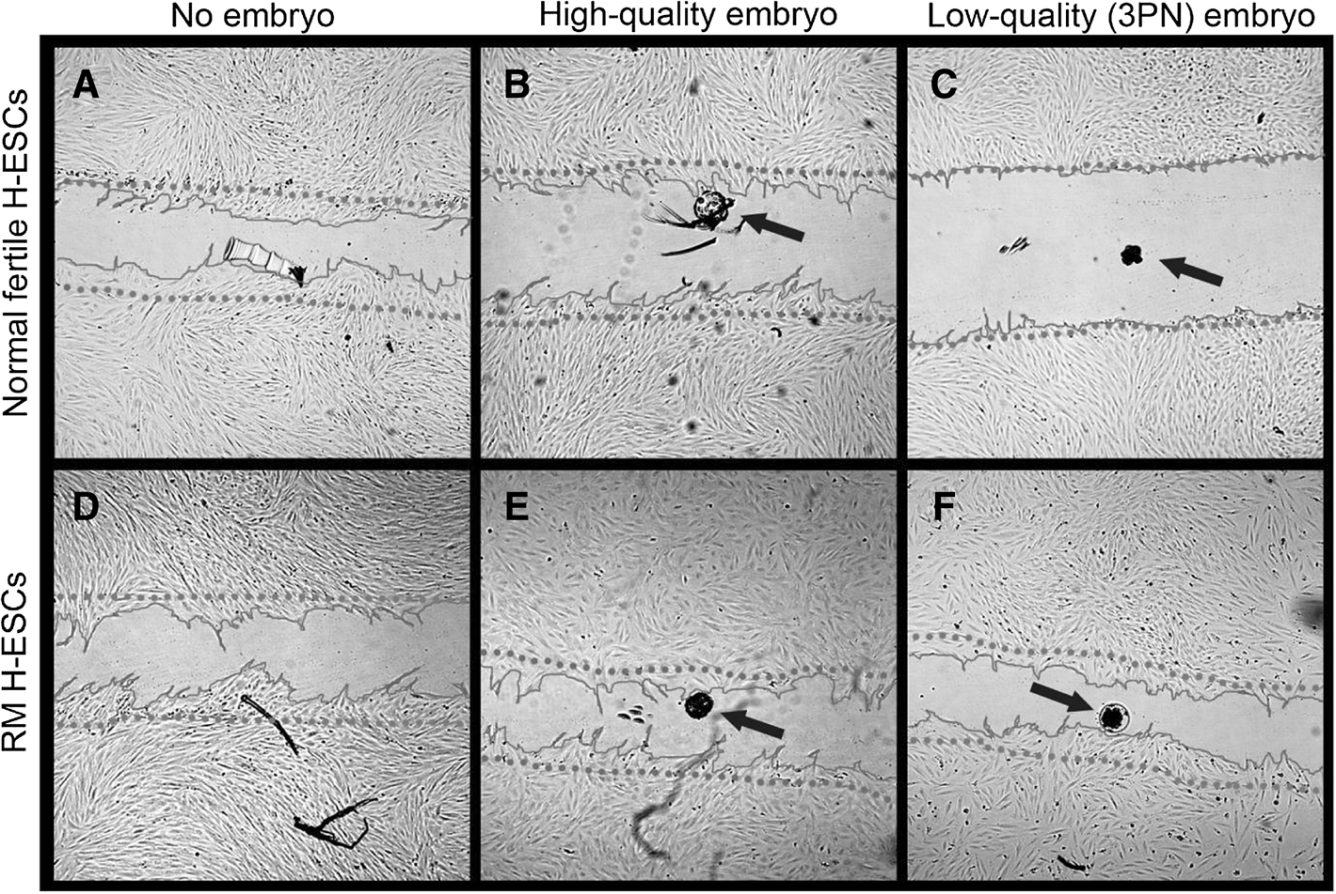
**The migration zone after adding a high-quality, low-quality or no embryo.** The migratory response of decidualized human embryonic neural stem cells (H-EnSCs) from normally fertile **(A-C)** and recurrent miscarriage (RM) women **(D-F)** was analyzed in absence of a human embryo (**A**,**D**), in presence of a high-quality embryo (B,E) or a low-quality embryo (**C**,**F**). Phase contrast pictures were taken 18 h after creating the migration zone. The dotted line represents the front of the migration zone directly after its creation. As a reference for the position of the embryo, the bottom of the plate was marked. The arrows indicate the position of the embryo. All pictures were taken with 25 × magnification. (Reproduced from Weimar *et al*. [26]).

Migration (scratch) assays have provided further evidence for altered embryo selectivity in women with RM. In a recent study, the directed migration of decidualized ESCs from normal fertile and RM women in the presence or absence of a high-quality or low-quality (chromosomally abnormal 3PN) embryo was observed [26]. The migration of ESCs from normal fertile women was totally inhibited in the presence of a low-quality embryo. However, the migration behavior of ESCs from women with RM was similar in the presence of both low-quality and high-quality embryos (Figure 2). In addition, in the presence of AC-1 M88 trophoblast cell-line-derived spheroids, the migration of ESCs from women with RM was enhanced compared to the normal fertile ESCs [26]. These observations suggest that ESCs from women with RM have an increased migratory potential in response to trophoblast signals and are more receptive (and thus less selective) for low-quality embryos than normally fertile women.

The clinical significance of these findings remains to be clarified, but failure of embryo selection may represent a single pathological pathway responsible for both euploidic and aneuploidic pregnancy losses. Brosens and Gellersen [65] have noted that given the high proportion of chromosomally abnormal preimplantation human embryos, this concept predicts that the likelihood of euploidic pregnancy failure increases with the number of miscarriages. This has indeed been shown to be the case [41].

This novel concept requires further elucidation and confirmation, but a growing body of evidence supports the notion of an active, selective decidual phenotype, which if disrupted may result in reproductive failure. Novel therapeutic options may thus be developed that can correct defects in selectivity, preventing inappropriate implantation of embryos of low viability and sparing women the severe stress caused by recurrent clinical miscarriage. Alternatively, *in vitro* fertilization and PGD may improve outcomes in this context as *in vitro* embryo selection would increase the chance of a viable embryo implanting. However, the efficacy of PGD in treating women with recurrent miscarriage due to translocations is unclear [54,66], and further studies are required.

### Uterine malformations

An accepted cause of recurrent pregnancy loss is uterine malformations that may be acquired or congenital. The latter include didelphic, bicornuate, arcuate, and septate uteri. In a recent systematic review including 89,861 women, the prevalence of all congenital uterine malformations was 5.5% (95% CI 3.5 to 8.5) in an unselected population and 15.4% (95% CI 10.3 to 23) among women with ≥3 miscarriages [27]. In another review comprising 3,805 women the meta-analysis showed that a septate uterus increased the rate of a first trimester miscarriage significantly when compared with women with normal uteri (RR = 2.89; 95% CI 2.02 to 4.14) [28]. The role of septal resection is being debated. Non-controlled studies suggest a positive effect on pregnancy outcome but we still lack prospective randomized trials [67].

### hCG gene polymorphisms and epigenetic causes

hCG is a glycoprotein composed of two subunits, α and β. Increasing amounts are secreted from the syncytiotrophoblast with increased gestation in the first trimester and bind to luteinizing hormone (LH)/hCG receptors on the corpus luteum preventing it from regression.

It is well known that early miscarriage is normally associated with low or suboptimally increasing hCG levels. The association between low hCG production and miscarriage can be interpreted in two ways: (1) the trophoblast growth may be delayed due to embryonal aneuploidy, immune or thrombophilic disturbances and low hCG production is a secondary phenomenon, or (2) the fetoplacental unit may secrete inadequate hCG due to a primary failure of the trophoblast to produce hCG, which will result in inadequate progesterone production and resulting embryonal death. Whereas the former condition in theory would not benefit from external hCG supplementation the latter condition may be treatable with external hCG or progesterone.

If the theory that some miscarriages are due to a primary failure of the trophoblast to produce hCG, the cause could be genetic. The β subunit of hCG is coded by four closely linked duplicate chorionic gonadotropin β (CGB) genes on chromosome 19, with CGB5 and CGB8 being the most active [68]. There is an association between levels of mRNA hCG-β transcripts in trophoblast tissue and plasma hCG levels and the levels of hCG-β mRNA seem to be lower in tissue from RM than from normal first trimester pregnancies or ectopic pregnancies. Specific polymorphisms in the promoter region of the CGB5 gene that may enhance hCG-β transcription have been found with lower prevalence in RM than in fertile couples [29] suggesting that some miscarriages in RM couples may be caused by polymorphism in the CGB genes. Couples with such polymorphisms may be those who would benefit from hCG supplementation, but this must be tested in prospective trials.

Recently, evidence has been presented suggesting that epigenetic disruptions may lie behind some instances of early pregnancy loss. During implantation embryos undergo demethylation and remethylation of DNA, which is crucial to their further development and health. In a study comparing methylation in embryos from medically terminated pregnancies with those from spontaneous losses, the villi derived from embryos lost in early pregnancy were found to express lower levels of DNA methyltransferase 1, an enzyme involved in maintaining methylation [69]. However, whether or not this is a causal rather than associated phenomenon with miscarriage remains to be elucidated. Numerous candidate genes associated with a small increase in the risk of early pregnancy loss have so far been described. The relative risk of miscarriage attributed to the carriage of most of these genetic polymorphisms is modest, dependent on the clinical and genetic background of the patients and screening for the polymorphisms is therefore not clinical useful [30,41,70].

### Lifestyle factors

Women experiencing sporadic as well as RM often have many questions regarding lifestyle factors. Although pregnant women are advised to refrain from alcohol a national Danish birth cohort study including nearly 100,000 pregnant women showed that 45% had some level of alcohol intake [31]. Even small amounts of alcohol increased the risk of a miscarriage significantly and further, the results suggested that the risk increased in a dose-related manner. Thus, the adjusted hazard ratio for a first trimester miscarriage was 1.66 and 2.82 when having 2 to 3.5 drinks per week and >4 drinks per week, respectively. In contrast to alcohol consumption, coffee drinking in pregnancy is fully acceptable in many countries. Another Danish study has looked into the association between miscarriage and coffee intake [71]. Only in cases where mothers were drinking more than seven cups of coffee a day could the authors demonstrate an increased risk of miscarriage (adjusted hazard ratio 1.48 (95% CI 1.01 to 2.17)).

Smoking-related complications in late pregnancy are substantial and well documented. In contrast, data are sparse and conflicting when it comes to smoking and miscarriage. As such, a recent review reports an increased risk of pregnancy loss among smokers [72] whereas a large prospective study including 24,608 pregnancies could not demonstrate an association between smoking and miscarriage [73].

There are many pregnancy-related complications associated with obesity, including miscarriage. A meta-analysis from 2008 including primarily studies on infertile populations showed significantly increased miscarriage rates when women with a body mass index (BMI) ≥25 kg/m^2^ were compared to women with a BMI <25 kg/m^2^[74]. This tendency has also been demonstrated in women with RM although it must be emphasized that a significantly increased risk of another miscarriage was demonstrated only in obese women; that is, BMI ≥30 kg/m^2^[32]. Interestingly, a logistic regression analysis showed that after advanced maternal age, increased BMI was the most important risk factor in predicting another miscarriage in women with RM.

A recent systematic review including 5 retrospective studies, 1 prospective study and a total cohort of nearly 30,000 women has investigated the relation between miscarriage rates and obesity (BMI ≥28 or 30 kg/m^2^) after spontaneous conception [33]. Indeed, they also found a significant association both as regards sporadic and RM implying an urgent need for prospective studies to assess the value of reducing BMI.

## Conclusions

Reproductive failure is a common complication in early pregnancy, with up to two-thirds of all fertilized oocytes not producing live births. Thus, a large number of conceptions either fail to implant or are categorized as biochemical pregnancies and clinical miscarriages. Although, the incidence of karyotypic abnormalities in the parents is low this high rate of early losses is most certainly connected to a high frequency of sporadic karyotypic abnormalities in the products of conception. In couples experiencing RM, however, a parental chromosomal anomaly is found ten times more frequently than in the background population and whether these couples should be offered PGD or await prenatal invasive diagnosis once a spontaneous pregnancy has been established is a matter under debate. Soon, sequencing of the entire fetal genome from free fetal DNA in the maternal circulation will be a standard procedure and will hopefully have the potential to increase our understanding of embryonic causes of both sporadic and RM.

Biomarkers associated with predisposition to thrombophilia or autoimmunity can be found with increased prevalence in women with RM and affect the prognosis negatively, but it is still unclear to what extent anticoagulation and immune modulation therapies can improve pregnancy outcome in these cases. Recent studies have highlighted the importance of genetically determined differences in capacity for hCG production and in markers of sperm DNA damage.

The emerging role of the endometrium as a biosensor of embryo quality, which may be less discerning in some women, also provides a novel mechanism underlying RM that merits further study. Sporadic miscarriage can be seen as representing nature’s quality control system, preventing embryos with severe abnormalities in most cases from progressing beyond the peri-implantation period. Should this quality control be disrupted, such embryos may be allowed to establish implantation long enough to present as clinical pregnancy before failing, resulting in recurrent clinical miscarriage. Clearly, if an embryo is of high quality, then having a less selective endometrium will not have clinical consequences, and an ongoing pregnancy may ensue. Consistent with this, most women with RM will achieve an ongoing pregnancy if they persist in trying.

However, other concurrent medical conditions outlined in this article may prevent the ready establishment of ongoing pregnancy. Recent and ongoing research is clarifying the varying mechanisms underlying the very distressing condition of RM and offer new opportunities for developing effective interventions.

## Abbreviations

BMI: Body mass index; ESC: Endometrial stromal cells; ESHRE: European Society of Human Reproduction and Embryology; hCG: Human chorionic gonadotropin; PCOS: Polycystic ovarian syndrome; PGD: preimplantation genetic diagnosis; RM: Recurrent miscarriage.

## Competing interests

The authors declare they have no competing interests.

## Authors’ contributions

NM, ECL and OBC made substantial contributions to conception and design. ECL, OBC, AMK, and NM contributed equally to the literature review, interpretation and writing of the manuscript. NM and OBC revised the manuscript critically. All authors read and approved the final manuscript.

## Authors’ information

ECL and OBC run the largest recurrent miscarriage clinic in Denmark. OBC is a senior member of the ESHRE Special Interest Group in Early Pregnancy Loss. NM has longstanding clinical and research interest in early pregnancy loss. All authors have published numerous papers and book chapters in this field.

## Pre-publication history

The pre-publication history for this paper can be accessed here:

http://www.biomedcentral.com/1741-7015/11/154/prepub

## References

[B1] Farquharson RG Jauniaux E Exalto N Updated and revised nomenclature for description of early pregnancy events Hum Reprod 2005 20 3008 3011 16006453 10.1093/humrep/dei167

[B2] Jauniaux E Farquharson RG Christiansen OB Exalto N Evidence-based guidelines for the investigation and medical treatment of recurrent miscarriage Hum Reprod 2006 21 2216 2222 16707507 10.1093/humrep/del150

[B3] Silver RM Branch DW Goldenberg R Iams JD Klebanoff MA Nomenclature for pregnancy outcomes: time for a change Obstet Gynecol 2011 118 1402 1408 22105271 10.1097/AOG.0b013e3182392977

[B4] Practice Committee of the American Society for Reproductive Medicine Evaluation and treatment of recurrent pregnancy loss: a committee opinion Fertil Steril 2012 98 1103 1111 22835448 10.1016/j.fertnstert.2012.06.048

[B5] Royal College of Obstetricians and Gynaecologists SAC Guideline No. 17. The investigation and treatment of couples with first and second trimester recurrent miscarriage 2011 London, UK: Royal College of Obstetricians and Gynaecologists 1 18

[B6] Holers VM Girardi G Mo L Guthridge JM Molina H Pierangeli SS Espinola R Xiaowei LE Mao D Vialpando CG Salmon JE Complement C3 activation is required for antiphospholipid antibody-induced fetal loss J Exp Med 2002 195 211 220 11805148 10.1084/jem.200116116 PMC2193604

[B7] Oku K Atsumi T Bohgaki M Amengual O Kataoka H Horita T Yasuda S Koike T Complement activation in patients with primary antiphospholipid syndrome Ann Rheum Dis 2009 68 1030 1035 18625630 10.1136/ard.2008.090670

[B8] King K Smith S Chapman M Sacks G Detailed analysis of peripheral blood natural killer (NK) cells in women with recurrent miscarriage Hum Reprod 2010 25 52 58 19819893 10.1093/humrep/dep349

[B9] Quenby S Nik H Innes B Lash G Turner M Drury J Bulmer J Uterine natural killer cells and angiogenesis in recurrent reproductive failure Hum Reprod 2009 24 45 54 18835875 10.1093/humrep/den348

[B10] Tang AW Alfirevic Z Quenby S Natural killer cells and pregnancy outcomes in women with recurrent miscarriage and infertility: a systematic review Hum Reprod 2011 26 1971 1980 21613313 10.1093/humrep/der164

[B11] Christiansen OB Kolte AM Dahl M Larsen EC Steffensen R Nielsen HS Hviid TV Maternal homozygocity for a 14 base pair insertion in exon 8 of the HLA-G gene and carriage of HLA class II alleles restricting HY immunity predispose to unexplained secondary recurrent miscarriage and low birth weight in children born to these patients Hum Immunol 2012 73 699 705 22537754 10.1016/j.humimm.2012.04.014

[B12] Robertson L Wu O Langhorne P Twaddle S Clark P Lowe GD Walker ID Greaves M Brenkel I Regan L Greer IA Thrombophilia in pregnancy: a systematic review Br J Haematol 2006 132 171 196 16398652 10.1111/j.1365-2141.2005.05847.x

[B13] McNamee K Dawood F Farquharson R Recurrent miscarriage and thrombophilia: an update Curr Opin Obstet Gynecol 2012 24 229 234 22729089 10.1097/GCO.0b013e32835585dc

[B14] Lassere M Empson M Treatment of antiphospholipid syndrome in pregnancy - a systematic review of randomized therapeutic trials Thromb Res 2004 114 419 426 15507273 10.1016/j.thromres.2004.08.006

[B15] Twig G Shina A Amital H Shoenfeld Y Pathogenesis of infertility and recurrent pregnancy loss in thyroid autoimmunity J Autoimmun 2012 38 J275 J281 22218218 10.1016/j.jaut.2011.11.014

[B16] van den Boogaard E Vissenberg R Land JA van Wely M van der Post JA Goddijn M Bisschop PH Significance of (sub)clinical thyroid dysfunction and thyroid autoimmunity before conception and in early pregnancy: a systematic review Hum Reprod Update 2011 17 605 619 21622978 10.1093/humupd/dmr024

[B17] Negro R Schwartz A Gismondi R Tinelli A Mangieri T Stagnaro-Green A Increased pregnancy loss rate in thyroid antibody negative women with TSH levels between 2.5 and 5.0 in the first trimester of pregnancy J Clin Endocrinol Metabol 2010 95 E44 E48 10.1210/jc.2010-0340 20534758

[B18] Cocksedge KA Saravelos SH Metwally M Li TC How common is polycystic ovary syndrome in recurrent miscarriage? Reprod Biomed Online 2009 19 572 576 19909600 10.1016/j.rbmo.2009.06.003

[B19] Robinson L Gallos ID Conner SJ Rajkhowa M Miller D Lewis S Kirkman-Brown J Coomarasamy A The effect of sperm DNA fragmentation on miscarriage rates: a systematic review and meta-analysis Hum Reprod 2012 27 2908 2917 22791753 10.1093/humrep/des261

[B20] Ribas-Maynou J Garcia-Peiro A Fernandez-Encinas A Amengual MJ Prada E Cortes P Navarro J Benet J Double stranded sperm DNA breaks, measured by Comet assay, are associated with unexplained recurrent miscarriage in couples without a female factor PLoS One 2012 7 e44679 23028579 10.1371/journal.pone.0044679 PMC3444447

[B21] Teklenburg G Salker M Molokhia M Lavery S Trew G Aojanepong T Mardon HJ Lokugamage AU Rai R Landles C Roelen BA Quenby S Kuijk EW Kavelaars A Heijnen CJ Regan L Brosens JJ Macklon NS Natural selection of human embryos: decidualizing endometrial stromal cells serve as sensors of embryo quality upon implantation PLoS One 2010 5 e10258 20422011 10.1371/journal.pone.0010258 PMC2858159

[B22] Quenby S Vince G Farquharson R Aplin J Recurrent miscarriage: a defect in nature’s quality control? Hum Reprod 2002 17 1959 1963 12151421 10.1093/humrep/17.8.1959

[B23] Aplin JD Hey NA Li TC MUC1 as a cell surface and secretory component of endometrial epithelium: reduced levels in recurrent miscarriage Am J Reprod Immunol 1996 35 261 266 8962658 10.1111/j.1600-0897.1996.tb00042.x

[B24] Salker M Teklenburg G Molokhia M Lavery S Trew G Aojanepong T Mardon HJ Lokugamage AU Rai R Landles C Roelen BA Quenby S Kuijk EW Kavelaars A Heijnen CJ Regan L Macklon NS Brosens JJ Natural selection of human embryos: impaired decidualization of endometrium disables embryo-maternal interactions and causes recurrent pregnancy loss PLoS One 2010 5 e10287 20422017 10.1371/journal.pone.0010287 PMC2858209

[B25] Teklenburg G Salker M Heijnen C Macklon NS Brosens JJ The molecular basis of recurrent pregnancy loss: impaired natural embryo selection Mol Hum Reprod 2010 16 886 895 20847090 10.1093/molehr/gaq079

[B26] Weimar CH Kavelaars A Brosens JJ Gellersen B de Vreeden-Elbertse JM Heijnen CJ Macklon NS Endometrial stromal cells of women with recurrent miscarriage fail to discriminate between high- and low-quality human embryos PLoS One 2012 7 e41424 22848492 10.1371/journal.pone.0041424 PMC3405140

[B27] Chan YY Jayaprakasan K Zamora J Thornton JG Raine-Fenning N Coomarasamy A The prevalence of congenital uterine anomalies in unselected and high-risk populations: a systematic review Hum Reprod Update 2011 17 761 771 21705770 10.1093/humupd/dmr028 PMC3191936

[B28] Chan YY Jayaprakasan K Tan A Thornton JG Coomarasamy A Raine-Fenning NJ Reproductive outcomes in women with congenital uterine anomalies: a systematic review Ultrasound Obstet Gynecol 2011 38 371 382 21830244 10.1002/uog.10056

[B29] Rull KCO Nagirnaja L Steffensen R Margus T Laan M A modest, but significant effect of CGB5 gene promotor polymorphisms in modulating the risk of recurrent miscarriage Fertil Steril 2013 99 1930 1936 23499152 10.1016/j.fertnstert.2013.02.019 PMC3698440

[B30] Daher S Mattar R Gueuvoghlanian-Silva BY Torloni MR Genetic polymorphisms and recurrent spontaneous abortions: an overview of current knowledge Am J Reprod Immunol 2012 67 341 347 22390536 10.1111/j.1600-0897.2012.01123.x

[B31] Andersen AM Andersen PK Olsen J Gronbaek M Strandberg-Larsen K Moderate alcohol intake during pregnancy and risk of fetal death Int J Epidemiol 2012 41 405 413 22253313 10.1093/ije/dyr189

[B32] Metwally M Saravelos SH Ledger WL Li TC Body mass index and risk of miscarriage in women with recurrent miscarriage Fertil Steril 2010 94 290 295 19439294 10.1016/j.fertnstert.2009.03.021

[B33] Boots C Stephenson MD Does obesity increase the risk of miscarriage in spontaneous conception: a systematic review Semin Reprod Med 2011 29 507 513 22161463 10.1055/s-0031-1293204

[B34] Zinaman MJ Clegg ED Brown CC O’Connor J Selevan SG Estimates of human fertility and pregnancy loss Fertil Steril 1996 65 503 509 8774277

[B35] Wilcox AJ Weinberg CR O’Connor JF Baird DD Schlatterer JP Canfield RE Armstrong EG Nisula BC Incidence of early loss of pregnancy N Engl J Med 1988 319 189 194 3393170 10.1056/NEJM198807283190401

[B36] Wang X Chen C Wang L Chen D Guang W French J Conception, early pregnancy loss, and time to clinical pregnancy: a population-based prospective study Fertil Steril 2003 79 577 584 12620443 10.1016/s0015-0282(02)04694-0

[B37] Macklon NS Geraedts JP Fauser BC Conception to ongoing pregnancy: the ‘black box’ of early pregnancy loss Hum Reprod Update 2002 8 333 343 12206468 10.1093/humupd/8.4.333

[B38] Nybo Andersen AM Wohlfahrt J Christens P Olsen J Melbye M Maternal age and fetal loss: population based register linkage study BMJ 2000 320 1708 1712 10864550 10.1136/bmj.320.7251.1708 PMC27416

[B39] Ugwumadu A Manyonda I Reid F Hay P Effect of early oral clindamycin on late miscarriage and preterm delivery in asymptomatic women with abnormal vaginal flora and bacterial vaginosis: a randomised controlled trial Lancet 2003 361 983 988 12660054 10.1016/S0140-6736(03)12823-1

[B40] Carp HJA Epidemiology of recurrent pregnancy loss Recurrent Pregnancy Loss 2007 London, UK: Informa Healthcare

[B41] Christiansen OB Steffensen R Nielsen HS Varming K Multifactorial etiology of recurrent miscarriage and its scientific and clinical implications Gynecol Obstet Invest 2008 66 257 267 18679035 10.1159/000149575

[B42] Philipp T Philipp K Reiner A Beer F Kalousek DK Embryoscopic and cytogenetic analysis of 233 missed abortions: factors involved in the pathogenesis of developmental defects of early failed pregnancies Hum Reprod 2003 18 1724 1732 12871891 10.1093/humrep/deg309

[B43] Vanneste E Voet T Le Caignec C Ampe M Konings P Melotte C Debrock S Amyere M Vikkula M Schuit F Fryns JP Verbeke G D’Hooghe T Moreau Y Vermeesch JR Chromosome instability is common in human cleavage-stage embryos Nat Med 2009 15 577 583 19396175 10.1038/nm.1924

[B44] Kurahashi H Tsutsumi M Nishiyama S Kogo H Inagaki H Ohye T Molecular basis of maternal age-related increase in oocyte aneuploidy Congenit Anom 2012 52 8 15 10.1111/j.1741-4520.2011.00350.x 22348779

[B45] Morris JK Wald NJ Watt HC Fetal loss in Down syndrome pregnancies Prenat Diagn 1999 19 142 145 10215072

[B46] Ogasawara M Aoki K Okada S Suzumori K Embryonic karyotype of abortuses in relation to the number of previous miscarriages Fertil Steril 2000 73 300 304 10685533 10.1016/s0015-0282(99)00495-1

[B47] Chiu RW Lo YM Clinical applications of maternal plasma fetal DNA analysis: translating the fruits of 15 years of research Clin Chem Lab Med 2013 1 197 204 23072857 10.1515/cclm-2012-0601

[B48] Kitzman JO Snyder MW Ventura M Lewis AP Qiu R Simmons LE Gammill HS Rubens CE Santillan DA Murray JC Tabor HK Bamshad MJ Eichler EE Shendure J Noninvasive whole-genome sequencing of a human fetus Sci Transl Med 2012 4 137 176 10.1126/scitranslmed.3004323 PMC3379884 22674554

[B49] Norton ME Brar H Weiss J Karimi A Laurent LC Caughey AB Rodriguez MH Williams J 3rd Mitchell ME Adair CD Lee H Jacobsson B Tomlinson MW Oepkes D Hollemon D Sparks AB Oliphant A Song K Non-Invasive Chromosomal Evaluation (NICE) Study: results of a multicenter prospective cohort study for detection of fetal trisomy 21 and trisomy 18 Am J Obstet Gynecol 2012 207 137 22742782 10.1016/j.ajog.2012.05.021

[B50] Dan S Wang W Ren J Li Y Hu H Xu Z Lau TK Xie J Zhao W Huang H Sun L Zhang X Liao S Qiang R Cao J Zhang Q Zhou Y Zhu H Zhong M Guo Y Lin L Gao Z Yao H Zhang H Zhao L Jiang F Chen F Jiang H Li S Wang J Clinical application of massively parallel sequencing-based prenatal noninvasive fetal trisomy test for trisomies 21 and 18 in 11,105 pregnancies with mixed risk factors Prenat Diagn 2012 32 1225 1232 23138752 10.1002/pd.4002

[B51] Branch DW Gibson M Silver RM Clinical practice. Recurrent miscarriage N Engl J Med 2010 363 1740 1747 20979474 10.1056/NEJMcp1005330

[B52] Franssen MT Korevaar JC van der Veen F Leschot NJ Bossuyt PM Goddijn M Reproductive outcome after chromosome analysis in couples with two or more miscarriages: index [corrected]-control study BMJ 2006 332 759 763 16495333 10.1136/bmj.38735.459144.2F PMC1420685

[B53] Franssen MT Korevaar JC Leschot NJ Bossuyt PM Knegt AC Gerssen-Schoorl KB Wouters CH Hansson KB Hochstenbach R Madan K van der Veen F Goddijn M Selective chromosome analysis in couples with two or more miscarriages: case–control study BMJ 2005 331 137 141 15985440 10.1136/bmj.38498.669595.8F PMC558698

[B54] Franssen MT Musters AM van der Veen F Repping S Leschot NJ Bossuyt PM Goddijn M Korevaar JC Reproductive outcome after PGD in couples with recurrent miscarriage carrying a structural chromosome abnormality: a systematic review Hum Reprod Update 2011 17 467 475 21504961 10.1093/humupd/dmr011

[B55] Lalioti MD Can preimplantation genetic diagnosis overcome recurrent pregnancy failure? Curr Opin Obstet Gynecol 2008 20 199 204 18460931 10.1097/GCO.0b013e3282f88e0c

[B56] Calleja-Agius J Jauniaux E Pizzey AR Muttukrishna S Investigation of systemic inflammatory response in first trimester pregnancy failure Hum Reprod 2012 27 349 357 22131390 10.1093/humrep/der402

[B57] Nielsen HS Steffensen R Varming K Van Halteren AG Spierings E Ryder LP Goulmy E Christiansen OB Association of HY-restricting HLA class II alleles with pregnancy outcome in patients with recurrent miscarriage subsequent to a firstborn boy Hum Mol Genet 2009 18 1684 1691 19223392 10.1093/hmg/ddp077

[B58] Hiby SE Regan L Lo W Farrell L Carrington M Moffett A Association of maternal killer-cell immunoglobulin-like receptors and parental HLA-C genotypes with recurrent miscarriage Hum Reprod 2008 23 972 976 18263639 10.1093/humrep/den011

[B59] Kruse C Rosgaard A Steffensen R Varming K Jensenius JC Christiansen OB Low serum level of mannan-binding lectin is a determinant for pregnancy outcome in women with recurrent spontaneous abortion Am J Obstet Gynecol 2002 187 1313 1320 12439525 10.1067/mob.2002.126846

[B60] Porter TF LaCoursiere Y Scott JR Immunotherapy for recurrent miscarriage Cochrane Database Syst Rev 2006 2 CD000112 10.1002/14651858.CD000112.pub2 16625529

[B61] Ata B Tan SL Shehata F Holzer H Buckett W A systematic review of intravenous immunoglobulin for treatment of unexplained recurrent miscarriage Fertil Steril 2011 95 1080 1085 21232738 10.1016/j.fertnstert.2010.12.021

[B62] Girardi G Redecha P Salmon JE Heparin prevents antiphospholipid antibody-induced fetal loss by inhibiting complement activation Nat Med 2004 10 1222 1226 15489858 10.1038/nm1121

[B63] Beshay VE Bukulmez O Sperm DNA damage: how relevant is it clinically? Curr Opin Obstet Gynecol 2012 24 172 179 22366964 10.1097/GCO.0b013e32835211b5

[B64] Grewal S Carver JG Ridley AJ Mardon HJ Implantation of the human embryo requires Rac1-dependent endometrial stromal cell migration Proc Natl Acad Sci USA 2008 105 16189 16194 18838676 10.1073/pnas.0806219105 PMC2562412

[B65] Brosens JJ Gellersen B Something new about early pregnancy: decidual biosensoring and natural embryo selection Ultrasound Obstet Gynecol 2010 36 1 5 20582930 10.1002/uog.7714

[B66] Fischer J Colls P Escudero T Munne S Preimplantation genetic diagnosis (PGD) improves pregnancy outcome for translocation carriers with a history of recurrent losses Fertil Steril 2010 94 283 289 20034626 10.1016/j.fertnstert.2009.02.060

[B67] Kowalik CR Goddijn M Emanuel MH Bongers MY Spinder T de Kruif JH Mol BW Heineman MJ Metroplasty versus expectant management for women with recurrent miscarriage and a septate uterus Cochrane Database Syst Rev 2011 6 CD008576 10.1002/14651858.CD008576.pub3 21678380

[B68] Rull K Laan M Expression of β-subunit of HCG genes during normal and failed pregnancy Hum Reprod 2005 20 3360 3368 16123088 10.1093/humrep/dei261 PMC1403819

[B69] Yin LJ Zhang Y Lv PP He WH Wu YT Liu AX Ding GL Dong MY Qu F Xu CM Zhu XM Huang HF Insufficient maintenance DNA methylation is associated with abnormal embryonic development BMC Med 2012 10 26 22413869 10.1186/1741-7015-10-26 PMC3355050

[B70] Chin JR Heuser CC Eller AG Branch DW Nelson LT Silver RM Leptin and leptin receptor polymorphisms and recurrent pregnancy loss J Perinatol In press 10.1038/jp.2013.25 23519368

[B71] Bech BH Nohr EA Vaeth M Henriksen TB Olsen J Coffee and fetal death: a cohort study with prospective data Am J Epidemiol 2005 162 983 990 16207803 10.1093/aje/kwi317

[B72] Saravelos SH Regan L The importance of preconception counseling and early pregnancy monitoring Semin Reprod Med 2011 29 557 568 22161468 10.1055/s-0031-1293209

[B73] Wisborg K Kesmodel U Henriksen TB Hedegaard M Secher NJ A prospective study of maternal smoking and spontaneous abortion Acta Obstet Gynecol Scand 2003 82 936 941 12956844 10.1034/j.1600-0412.2003.00244.x

[B74] Metwally M Ong KJ Ledger WL Li TC Does high body mass index increase the risk of miscarriage after spontaneous and assisted conception? A meta-analysis of the evidence Fertil Steril 2008 90 714 726 18068166 10.1016/j.fertnstert.2007.07.1290

